# Dynapenia, Muscle Quality, and Hepatic Steatosis in Patients with Obesity and Sarcopenic Obesity

**DOI:** 10.3390/biomedicines11020472

**Published:** 2023-02-06

**Authors:** Francesco Frigerio, Maria De Marinis, Francesca Camardella, Vito Cantisani, Alessandro Pinto, Marco Bernardi, Carla Lubrano, Lucio Gnessi, Massimo Federici, Lorenzo Maria Donini, Eleonora Poggiogalle

**Affiliations:** 1 Department of Experimental Medicine, Sapienza University, P.le Aldo Moro 5, 00185 Rome, Italy; 2 Department of Radiological, Oncological and Pathobiological Sciences, Sapienza University of Rome, 00144 Rome, Italy; 3 Department of Clinical, Internal Medicine, Anesthesiology and Cardiovascular Sciences, Sapienza University, P.le Aldo Moro 5, 00185 Rome, Italy; 4 Department of Systems Medicine, University of Rome Tor Vergata, Center for Atherosclerosis, Policlinico Tor Vergata, Via Montpellier, 1, 00133 Rome, Italy

**Keywords:** sarcopenic obesity, handgrip strength, fatty liver disease, muscle quality, NAFLD, insulin resistance, hepatic steatosis, dynapenia

## Abstract

Accumulating evidence supports a connection between sarcopenic obesity (SO) and NAFLD. The extent to which fatty liver contributes to impaired muscle contractility is not yet well established. The aim of our study was to investigate the effect of NAFLD on dynapenia in patients with SO. In this study, 71 non-diabetic subjects (age 55 (7.8) years, BMI 35.2 kg/m^2^ (32.6–38.8)) were classified as having SO and non-sarcopenic obesity (NSO). SO patients displayed worse serum lipid profiles, higher body fat, and lower skeletal muscle mass (both total and appendicular) than NSO patients, despite the absence of any significant differences in body weight, glycometabolic parameters, and hepatic steatosis prevalence. A positive correlation between disposition index and muscle quality index (MQI) (*r* = 0.393, *p* = 0.013) emerged after controlling for menopause and body fat percentage. Based on multiple linear regression analysis, MQI was significantly positively associated with the disposition index (β: 0.059, SE: 0.002, *p* = 0.006) after adjustment for menopause, body fat percentage, and the presence of hepatic steatosis according to the hepatorenal index (HRI). Similar findings emerged when including liver enzyme levels in place of hepatic steatosis. Muscle quality was positively associated with β-cell function corrected for insulin resistance among patients with obesity and sarcopenic obesity, irrespective of the presence of fatty liver disease.

## 1. Introduction

Recently, mounting evidence has accumulated in the field of sarcopenic obesity, due to the epidemiological connections between the parallel increasing trends in both aging and obesity [1]. Chronic excess energy exposure is responsible for the development of overweight and obesity, as well as being the origin of ectopic fat deposition, leading to a number of metabolic complications, with nonalcoholic fatty liver disease (NAFLD) as one of the most frequent comorbidities of obesity [2]. The overall prevalence of NAFLD worldwide has been estimated to be 32.4% (95% CI 29.9–34.9), showing an approximately 1.5-fold increase in the last two decades in the general adult population [3]. Alterations in body composition occurring in the phenotype of sarcopenic obesity over the life course—namely, the increase in body fat and the decline in lean body mass, and lipid accumulation in the hepatocytes—stem from a common matrix of pathogenetic mechanisms, mainly encompassing insulin resistance and inflammation, along with other hormonal derangements [4]. A growing body of evidence supports a positive association between sarcopenic obesity and NAFLD, with even higher risk estimates for hepatic steatosis than either sarcopenia or obesity alone [5,6]. Furthermore, especially in the elderly, in recent years accumulating evidence has highlighted the novel relationship between fatty liver and the decrease in muscle strength (i.e., dynapenia) that represents another signature of the sarcopenic phenotype [7]. In a large Thai cohort of individuals with NAFLD, poor muscle strength was independently associated with long-term overall mortality. Adult and elderly men in the highest fatty liver index (FLI) quartile were sixfold more likely to have sarcopenia and had lower mean radial and tibial muscle densities measured using pQCT when compared to the lowest quartile [8,9]. Similarly, a Korean study found that middle-aged men (>50 years old) and postmenopausal women in the highest quartile of the hepatic steatosis index (HSI) had a 5.63- and 3.58-fold higher risk of having reduced muscle strength, respectively, compared to their counterparts in the lowest quartile [10]. Actually, the decrease in lean mass does not thoroughly explain the reduction in muscle strength, the latter dropping at a faster rate than the decline in skeletal muscle mass [11]. The extent to which the presence of fatty liver can contribute to impaired muscle contractility and strength generation in patients with sarcopenic obesity is not yet well established. The aim of the present study was to investigate the potential role played by hepatic steatosis in the context of dynapenia and reduced muscle quality in adult patients with sarcopenic obesity.

## 2. Materials and Methods

Study participants were recruited among patients referred to the High Specialization Center for the Care of Obesity, Policlinico “Umberto I” Hospital, Sapienza University, Rome, Italy. The inclusion criteria were as follows: age > 18 and <75 years, body mass index ≥ 30 kg/m^2^, Caucasian ethnicity. The exclusion criteria were as follows: malignant disease (last 5 years), inflammatory or autoimmune diseases, corticosteroids for systemic use, medication potentially affecting body weight/composition, syndromic obesity, renal failure (GFR < 60 mL/min), heart failure (NYHA classes III–IV), diabetes, history of viral or autoimmune liver disease or any other chronic liver disease, alcohol intake > 140 g/week for men and 70 g/week for women, participation in a weight-reducing program (last 3 months), participation in a physical exercise program, therapy (last 6 months) with antibiotics, bile salts, cholestyramine, previous cholecystectomy, gallbladder disease, any neurodegenerative diseases, or any musculoskeletal diseases. The study protocol was approved by the Ethical Committee of the “Sapienza” University, Rome, Italy. Written informed consent was obtained from all participants. All subjects underwent a complete physical examination; assessment of past medical history and medications was also performed. **Anthropometric measurements**. Body weight, height, and waist circumference were measured following standardized procedures [12]. The same tools were used in all subjects: a weighing scale (SECA 700, SECA, Hamburg, Germany) to the nearest 0.1 kg, a flexible tape (SECA 200, SECA, Hamburg, Germany) to the nearest 0.1 cm and a telescopic stadiometer (SECA 217, SECA, Hamburg, Germany) to the nearest 0.1 cm. Body mass index (BMI) was calculated as body weight (kg) divided by height squared (m^2^), while waist-to-height ratio (WHtR) as the ratio of waist circumference (cm) and height (cm). **Definition of obesity and sarcopenic obesity.** Obesity was defined as BMI ≥ 30 kg/m^2^. Sarcopenic obesity was defined in accordance with the 2022 EASO-ESPEN consensus statement [13]. **Body composition analysis**. Absolute and relative fat mass (FM, body fat percentage), absolute and relative fat-free mass (FFM, % FFM) and appendicular skeletal muscle mass (i.e., upper arms) were assessed by segmental multi-frequency bioimpedance analysis (S-MFBIA) (SECA mBCA 525, SECA, Hamburg, Germany). Whole-body skeletal muscle mass (total SMM) was estimated according to the equation presented by Jannsen [14]. **Biochemistry and derived indices**. Blood collection was performed after a 10–12 h overnight fast. Commercial kits were used to assay the following biochemical analytes: aspartate aminotransferase (AST), alanine aminotransferase (ALT), γGT (gamma-glutamyl transpeptidase), total cholesterol (TOT-C), HDL cholesterol (HDL-C), LDL cholesterol (LDL-C), triglyceride (TG), basal glucose (Glu_0′_), basal insulin (Ins_0′_), creatinine, and urate. Homeostasis model assessment of insulin resistance (HOMA-IR)] [15] and oral disposition index [16] were calculated in order to evaluate the presence of insulin resistance, alone or corrected for pancreatic β-cell function, respectively (see references for further details). After collecting fasting samples, a standard 75 g OGTT was performed, and blood samples were withdrawn after 2 h; 2-h glucose and insulin (Glu_120′_ and Ins_120′_) were measured using commercial kits. **Muscle strength and quality**. Study participants underwent the handgrip strength (HGS) test [17,18] using a digital dynamometer (DynEx, Akern, Florence, Italy). The arithmetic mean of three consecutive measurements was calculated for each arm. Moreover, the mean value obtained was normalized to the SMM of the homolateral arm, similar to previous studies [19], and was used as a proxy for muscle quality (namely, muscle quality index—MQI) [20,21]. **Definition of physical activity level**. Patients completed the self-administered International Physical Activity Questionnaire Short Form (IPAQ-SF); physical activity levels were categorized into low, moderate, and high in accordance with the Guidelines for Data Processing and Analysis of the International Physical Activity Questionnaire (IPAQ) (www.ipaq.ki.se; see also [22]). **Hepatic ultrasonography**. Participants were asked to fast for at least six hours before the B-mode ultrasound. An experienced radiologist performed a conventional grayscale ultrasound with QUS imaging using a clinical US system (Samsung RS85A Prestige, Samsung Healthcare, Sacramento, CA, USA) with a convex probe. Conventional B-mode US images of the liver were obtained using subcostal and intercostal planes and stored. Hepatic steatosis was evaluated based on a semi-quantitative method, i.e., the computerized calculation of the hepatorenal index. Studies have shown that the hepatorenal index (HRI) or hepatorenal ratio (HRR) is a sensitive and non-invasive test [23,24,25]. It is a simple calculation of the B-mode ratio, or the brightness ratio of the liver parenchyma over the renal cortex in each user-selected ROI. EzHRI™ functions in much the same way as the conventional HRI but offers greater convenience and an improved workflow by suggesting initial ROI positions. The calculation involved in determining initial ROI positions requires three steps: Liver and kidney segmentation, ROI extraction from both the liver and the kidney on a user-selected image, and calculation of the HRI. EzHRI™ segments the input image into the kidneys and liver, based on deep learning technology. It then uses the stochastic analysis method, which extracts three brightness classes from each segmented organ, to extract only the cortex while excluding other anatomical structures and image artifacts. Finally, it finds the ROIs exhibiting the lowest brightness variance in the liver and kidneys, calculates the average brightness ratio for each ROI, and then measures the HRI value. Users may also position the ROIs based on individual preferences and measure the HRI value. EzHRI™ provides users with a quantified HRI. Hepatic steatosis was detected when the HRI was ≥1.28 [26]. **Surrogate indices of visceral adiposity, hepatic steatosis, and hepatic fibrosis.** The visceral adiposity index (VAI) [27], lipid accumulation product (LAP) [28], fatty liver index (FLI) [29], fibrosis-4 score (FIB-4) [30], and NAFLD fibrosis score (NFS) [31] were also computed according to their respective formulae (see references for further details). **Statistical analysis**. The model assumptions were verified before conducting each of the following data analyses. Distributions of continuous variables were examined for skewness and kurtosis, and they were numerically transformed when appropriate to adjust their distributional patterns using logarithmic, square root, or reciprocal functions. Transformed variables are presented as untransformed values for ease of reading. Due to the unbalanced design of the sarcopenic obese (SO) and non-sarcopenic obese (NSO) groups, the unequal variance *t*-test (Welch’s *t*-test) was used for comparing the means of normally distributed variables; the Mann–Whitney U test was used to compare the distributions of non-normally distributed variables. As for differences in proportions, the Wald test was employed. After removing univariate outliers, a simple scatterplot and Pearson’s product–moment correlation coefficient (*r*) were used to examine the linear relationships between left or right MQI and disposition index; after controlling for menopause and body fat percentage, partial correlation coefficients were also computed (partial *r*). Multiple linear regression analysis was employed to examine the associations between muscle strength/quality and the variables included in the models. The covariates included in the models were chosen a priori from among those factors expected to influence the dependent variable, based on biological mechanisms or evidence from research; they are specified in the Section 3. The level of significance for all statistical tests was set at *p* < 0.05. Data analyses were performed using SPSS (IBM SPSS Statistics for Windows Version 27.0, IBM Corp, Armonk, NY, USA).

## 3. Results

A total of 71 patients [age 55 (7.8) years, BMI 35.2 kg/m^2^ (32.6–38.8)] were included in the final analysis; women represented 66.2% of the whole sample (*n* = 47). After grouping the subjects into sarcopenic obese (SO) and non-sarcopenic obese (NSO) groups, we tested for differences in key clinical variables (Table 1). Despite the reduced mean body weight in the SO group (*p* < 0.05), with no differences in waist circumference and WHtR, body composition analysis by S-MFBIA showed higher body fat percentage and reduced FFM and total SMM (all *p* < 0.01). Likewise, the HGS values (absolute or normalized to homolateral arm SMM as MQI) were significantly reduced in the SO patients (all *p* < 0.01). No differences were apparent in terms of hepatic steatosis prevalence and surrogate indices of hepatic steatosis and fibrosis; moreover, glycometabolic parameters and β-cell function corrected for insulin resistance (disposition index) were comparable between the two groups. Higher levels of TOT-C, and LDL-C (*p* < 0.05) were seen among SO patients, with a trend for significance for higher HDL-C (*p* = 0.051) despite similar use of hypolipidemic agents. Finally, blood pressure (SBP and DBP) values appeared to be similar for SO and NSO patients, which consistent with comparable levels of physical activity and antihypertensive treatment use.

**Figure 1:**
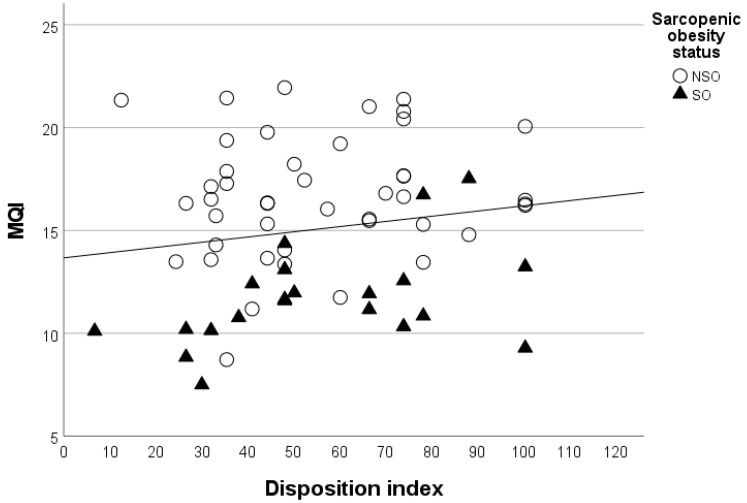
Relationship between left muscle quality index (MQI) and disposition index. Distribution of left MQI and disposition index values among non-sarcopenic obese (NSO) patients (empty white circles) and sarcopenic obese (SO) patients (full black triangles). All *r* = 0.409, *p* = 0.01 (see text for further details).

We investigated potential correlations between body composition, muscle function and insulin resistance parameters. The relationship between left MQI and disposition index, grouped by sarcopenic obesity status, is depicted in Figure 1; according to the partial correlation analysis, after controlling for body fat percentage and menopause, a positive correlation was apparent between the disposition index and left MQI (partial *r* = 0.409, *p* = 0.01), but not right MQI (partial *r* = 0.245, *p* = 0.133).

Based on multiple linear regression analysis, the disposition index was positively associated with MQI (β: 0.059, SE: 0.002, *p* = 0.006) after adjustment for the presence of hepatic steatosis, menopause, and body fat percentage (Table 2). When replacing the presence of hepatic steatosis according to the HRI with ALT levels, the positive association between the disposition index and MQI remained significant (β: 0.069, SE: 0.022, *p* = 0.004), but no significant association was found between ALT concentrations and MQI (Table 3) Analogous findings were obtained when including γGT levels in place of either hepatic steatosis or ALT concentrations (β: 0.065, SE: 0.023, *p* = 0.008) (Table 4). Finally, a model including only body fat percentage and menopause did not reach statistical significance (Table 5).

## 4. Discussion

In the present study, the prevalence of hepatic steatosis according to the HRI cutoff point was not significantly different between sarcopenic and non-sarcopenic obese subjects. A number of studies provided different cutoff values for the HRI; we relied on a threshold obtained from cases of hepatic steatosis rather than chronic liver diseases—less specific for the presence of fatty liver. We observed a positive association between the muscle quality index (MQI) and the disposition index—as proxies of muscle strength and insulin sensitivity, respectively—after adjustment for menopause, body fat percentage, and the presence of hepatic steatosis. This association was confirmed when considering either ALT concentrations or GGT levels as covariates in place of the fatty liver, based on ultrasonography; neither fatty liver nor liver enzyme levels were found to be significantly associated with the MQI. Thus, in patients with obesity and sarcopenic obesity, muscle quality was positively associated with insulin sensitivity per se, irrespective of the presence of fatty liver. Our findings showing that dynapenia was negatively associated with the disposition index, regardless of the presence of hepatic steatosis, are in disagreement with prior studies highlighting an inverse relationship between NAFLD and muscle strength. In a study by Kim et al., handgrip strength was found to be reduced in a cohort of Korean middle-aged and elderly men and women with NAFLD (based on the hepatic steatosis index) after controlling for potential confounding factors, such as body weight, blood lipid profile, and glycated hemoglobin (HbA1c), but no indicator of insulin resistance was included in the model [32]. Conversely, in a large Chinese cohort, low muscle strength was associated with NAFLD (defined by the presence of ≥2 of the following abdominal US signs: diffusely increased liver echogenicity, vascular blurring, and deep attenuation [33]) after accounting for a surrogate index of insulin resistance (i.e., HOMA-IR, OR: 1.18) and other confounding covariates [5]. Similar to the abovementioned Asian studies, in a European cohort of more than 300,000 participants, those in the middle or the highest tertile of grip strength exhibited a lower risk of severe NAFLD than their counterparts in the lowest tertile (0.82- (95% CI 0.76–0.90) and 0.70-fold (95% CI 0.64–0.77), respectively). In that study, the diagnosis of either severe NAFLD or nonalcoholic steatohepatitis (NASH) was retrieved from hospital and death databases, and the analyses were adjusted for confounding factors, including the components of metabolic syndrome, but insulin resistance was not accounted for [7]. These inconsistencies may be due to the different methods used for assessing the presence of hepatic steatosis, as well as the heterogeneity in the study populations in terms of age and ethnicity groups. Indeed, another relevant factor accounting for the discrepant findings is represented by the acknowledgement of insulin resistance or insulin sensitivity as explanatory covariates mediating the association between muscle strength and NAFLD. On the one hand, skeletal muscle insulin resistance is a well-known mechanism with a detrimental impact on skeletal muscle contractility, especially due to the accumulation of lipotoxic species within the muscle fibers concomitant with exposure to a high-fat diet [34]. Moreover, a number of studies on dynapenia and type 2 diabetes further support the relevance of metabolic derangements related to insulin sensitivity and glucose tolerance in the decline of muscle strength, especially when dynapenia is coupled with abdominal obesity [35,36]. Previously, we showed a negative association between dynapenia and insulin resistance in women with metabolic syndrome [19]. On the other hand, insulin resistance is also considered to be the pathophysiological phenomenon underpinning the development of nonalcoholic fatty liver disease [37,38]. Hence, this may enlighten our findings, with the disposition index as the relevant explanatory variable for the alterations in muscle strength and quality, and with hepatic steatosis not playing a major role in the regression analysis. These observations are plausible and capture the potential role of altered insulin sensitivity as a common condition underlying the phenotypes of both dynapenia and fatty liver. Several limitations and strengths of the present study need to be acknowledged. One limitation of our study is the inclusion of subjects with a BMI ≥ 30 kg/m^2^, which could partially explain the high prevalence of hepatic steatosis and, thus, its marginal role as a predictor of MQI in our regression model, likely supposing a ceiling effect. However, the use of quantitative ultrasound (i.e., HRI) in place of qualitative assessment and the adoption of the current EASO-ESPEN definition of sarcopenic obesity could also explain the discrepancy with previous studies showing an inverse relationship between liver fat content and handgrip strength. Moreover, using arm SMM from S-MFBIA to normalize handgrip strength could represent a more physiological proxy of muscle quality compared to body mass or total SMM. Further limitations are the small sample size and gender imbalance (i.e., higher proportion of women) in the NSO and SO groups; although it could have overshadowed baseline differences in glucose and lipid profile, the purported lower cardiometabolic risk among women has been disputed [39]. Moreover, in both groups most of the female patients were in the menopause state—a non-modifiable risk factor per se. Finally, we could not evaluate liver fibrosis status by means of radiological techniques or liver biopsy (the current gold-standard); although we used validated serum markers (i.e., FIB-4 and NFS), we cannot exclude the presence of non-advanced liver fibrosis [40,41].

In conclusion, muscle quality is positively associated with β-cell function corrected for insulin resistance among patients with obesity and sarcopenic obesity, irrespective of the presence of fatty liver disease. In line with the EASO-ESPEN consensus on sarcopenic obesity, functional status should be implemented for an exhaustive assessment of metabolic derangements among subjects with obesity. Moreover, the combined use of segmental BIA for ASMM and hand dynamometer for HGS seems promising in this specific cohort of patients, since they are both available in everyday clinical practice and allow the computation of a proxy variable of impaired skeletal muscle functionality, i.e., the muscle quality index. Further research should be prompted in order to thoroughly clarify the role of fatty liver infiltration in the onset and development of functional alterations, such as dynapenia, in the context of sarcopenic obesity.

## Figures and Tables

**Table 1:** Baseline characteristics of non-sarcopenic obese (NSO) and sarcopenic obese (SO) subjects.

	Variable (Units)	NSO Subjects (*n* = 49)	SO Subjects (*n* = 22)	*p*-Value
Demographics	Age (years)	52.2 (8)	56.2 (5.9)	*
	Females (N, %)	29, 59.2%	18, 81.8%	0.074
	Menopause (N, %)	18, 62.1%	15, 83.3%	ns
Anthropometry and body composition	Weight (kg)	97.6 (88.1–107.0)	88.1 (74.6–106.3)	*
	BMI (kg/m^2^)	35.32 (32.86–38.30)	34.64 (31.93–40.16)	ns
	Waist (cm)	111.7 (104.2–122.3)	107.8 (97.9–118.5)	ns
	WHtR	0.95 (0.91–1.02)	0.93 (0.92–0.98)	ns
	FM (kg)	41.6 (35.8–49.7)	41.3 (35.3–48.3)	ns
	Body fat (%)	42.6 (39.4–47.9)	48.6 (44.9–50.0)	**
	FFM (kg)	54.8 (47.3–65.8)	45.2 (38.3–52.5)	**
	Total SMM (kg)	21.9 (24.5–32.5)	19.7 (17.7–24.8)	**
	Total SMM/weight	0.266 (0.236–0.305)	0.239 (0.219–0.262)	*
Muscle strength and derived indices	Left HGS (kg)	28.6 (20.8–33.7)	13.7 (12.4–16.4)	**
	Right HGS (kg)	28.1 (21.1–35.0)	15.6 (12.6–16.8)	**
	Left MQI	16.5 (15.3–19.4)	11.4 (10.2–12.7)	**
	Right MQI	17.5 (15.5–20.0)	12.0 (10.5–14.5)	**
Glucose–insulin metabolism	Glu_0′_ (mg/dL)	94 (86–103)	95 (86–106)	ns
	Glu_120′_ (mg/dL)	105 (82–126)	95 (67–110)	0.075
	Ins_0′_ (mUI/mL)	16.0 (11.4–23.5)	13.4 (10.1–20.2)	ns
	Ins_120′_ (mUI/mL)	90.6 (40.8–145.6)	60.8 (20.2–96.2)	ns
	HbA1c (mmol/mol)	35.0 (33.8–38.0)	37.0 (36.0–39.5)	*
	Disposition index	49.1 (35.5–74.0)	48.1 (35.1–76.1)	ns
Lipid profile and visceral adiposity indices	TOT-C (mg/dL)	189 (175–213)	229 (192–252)	**
	LDL-C (mg/dL)	121 (100–135)	146 (128–173)	*
	HDL-C (mg/dL)	49 (41–59)	53 (46–67)	0.051
	TG (mg/dL)	118 (72–154)	112 (87–133)	ns
	LAP	61.0 (39.1–94.1)	60.0 (44.8–72.3)	ns
	VAI	94.2 (61.8–144.8)	107.4 (78.6–136.0)	ns
Clinical biochemistry	AST (U/L)	19 (16–24)	19 (15–20)	ns
	ALT (U/L)	19.5 (14.3–37.5)	16.5 (13.3–22.0)	*
	γGT (U/L)	18.5 (13.8–35.3)	18.5 (13.8–35.3)	*
	Creatinine (mg/dL)	0.86 (0.74–0.97)	0.80 (0.78–0.92)	ns
	Urate (mmol/L)	5.0 (1.4–5.7)	5.5 (4.8–6.0)	*
Liver steatosis and fibrosis indices	FLI	85.6 (69.3–94.0)	73.7 (43.4–94.0)	ns
	FIB-4	0.92 (0.68–1.27)	0.97 (0.77–1.21)	ns
	NFS	−1.02 (−1.9, −0.08)	−0.68 (−2.0, −0.12)	ns
Liver steatosis	N, %	34, 69.4%	10, 45.5%	0.098
Physical activity level	Low (N, %)	25, 51.0%	11, 50.0%	ns
	Moderate (N, %)	16, 32.7%	9, 40.9%	ns
	High (N, %)	8, 16.3%	2, 9.1%	ns
Medications	Antihypertensive N, %	17, 35.4%	9, 40.9%	ns
	Hypolipidemic N, %	8, 16.7%	1, 4.5%	ns

Results are displayed as the mean (standard deviation) for normally distributed variables and the median (1st quartile–3rd quartile) for non-normally distributed variables; * *p* < 0.05 for intergroup difference; ** *p* < 0.01 for intergroup difference; ns = not significant (*p* ≥ 0.1). Abbreviations: ALT = alanine aminotransferase; AST = aspartate aminotransferase; BMI = body mass index; FFM = fat-free mass; FIB-4 = fibrosis-4 score; FLI = fatty liver index; FM = fat mass; Glu_0′_ (mg/dL) = basal glycemia; Glu_120′_ = 2-h glucose; HbA1c = glycosylated hemoglobin; HDL-C = high-density lipoprotein cholesterol; HGS = handgrip strength; Ins_0′_ = basal insulin; Ins_120′_ = 2-h insulin; LAP = lipid accumulation product; LDL-C = low-density lipoprotein cholesterol; MQI = muscle quality index; NFS = NAFLD fibrosis score; SMM = skeletal muscle mass; TG = triglycerides; TOT- C = total cholesterol; VAI = visceral adiposity index; WHtR = waist-to-height ratio; γGT (U/L) = gamma-glutamyl transpeptidase.

**Table 2:** Model 1—MQI as the outcome variable using body fat percentage, menopause, disposition index, and hepatic steatosis as predictors. LLC = lower level of confidence; ULC = upper level of confidence.

Model		Unstandardized Coefficients		Standardized Coefficients	*t*	Sig.	95% C.I. for B	
		B	Std. Error	Beta			LLC	ULC
1	Intercept	10.902	5.781		1.886	0.067	−0.822	22.626
	Body fat	−0.008	0.116	−0.011	−0.071	0.944	−0.244	0.228
	Menopause	−1.056	1.119	−0.142	−0.944	0.352	−3.324	1.213
	Disposition index	0.059	0.02	0.425	2.927	0.006	0.018	0.100
	Hepatic steatosis	1.911	1.002	0.279	1.907	0.064	−0.121	3.943

**Table 3:** Model 2—Outcome variable: MQI. Predictors: body fat percentage, menopause, disposition index, and γGT levels.

Model		Unstandardized Coefficients		Standardized Coefficients	*t*	Sig.	95.0% C.I. for B	
		B	Std. Error	Beta			LLC	ULC
2	Intercept	11.134	6.075		1.833	0.076	−1.256	23.523
	Body fat	0.021	0.128	0.027	0.163	0.872	−0.241	0.283
	Menopause	−1.797	1.340	−0.235	−1.341	0.190	−4.529	0.936
	Disposition index	0.065	0.023	0.469	2.853	0.008	0.019	0.112
	γGT	−0.015	0.043	−0.063	−0.352	0.727	−0.104	0.073

**Table 4:** Model 3—Outcome variable: MQI. Predictors: body fat percentage, menopause, disposition index, and ALT levels.

Model		Unstandardized Coefficients		Standardized Coefficients	*t*	Sig.	95% C.I. for B	
		B	Std. Error	Beta			LLC	ULC
3	Intercept	11.384	6.204		1.835	0.076	−1.252	24.021
	Body fat	−0.027	0.122	−0.035	−0.224	0.824	−0.275	0.220
	Menopause	−1.230	1.258	−0.155	−0.978	0.336	−3.792	1.332
	Disposition index	0.069	0.022	0.496	3.144	0.004	0.024	0.114
	ALT	0.061	0.057	0.170	1.076	0.290	−0.055	0.177

**Table 5:** Model 4—Outcome variable: MQI. Predictors: body fat percentage and menopause.

Model		Unstandardized Coefficients		Standardized Coefficients	t	Sig.	95% C.I for B	
		B	Std. Error	Beta			LLC	ULC
4	Intercept	17.538	5.961		2.942	0.006	5.469	29.606
	Body fat	−0.043	0.127	−0.056	−0.338	0.738	−0.300	0.214
	Menopause	−1.455	1.228	−0.195	−1.185	0.243	−3.942	1.031

## Data Availability

Data are available to third parties upon request addressed to the corresponding author.
